# Evaluation of utility and usefulness of webinars on COVID-19 management: a questionnaire-based survey

**DOI:** 10.1186/s42077-021-00187-x

**Published:** 2021-10-20

**Authors:** Rekha Gupta, B. Naveen Naik, Venkata Ganesh, Ajay Singh, Shiv Lal Soni, G. D. Puri

**Affiliations:** 1 Department of Anaesthesia & Intensive Care, Post Graduate Institute of Medical Education and Research, Sector 12, Chandigarh, 160012 India; 2 Department of Anaesthesia & Intensive Care, Post Graduate Institute of Medical Education and Research, Chandigarh, 160012 India

**Keywords:** COVID-19, Webinars, Healthcare workers

## Abstract

**Background:**

The COVID-19 pandemic and its consequent “social distancing” has fueled the use of social media platforms for educational purposes. Since the start of the pandemic, a plethora of experts and self-proclaimed experts have been keenly delivering webinars on COVID. This begs the question “Do webinars on COVID-19 really help in the improvement of knowledge base or management skills?”. The questionnaire was designed to assess information regarding COVID-webinars and their usefulness from the end-user standpoint. The response to the questions was measured using a 4- or 5-point Likert scale. The survey was open for a 4-week period with the extension of 1 week.

**Results:**

The response rate was 54% as 270 out of 500 participants responded to the questionnaire. The majority of the respondents were anesthesiologists in-training, post-graduates, fellows, and seniors belonging to tertiary care settings with fewer percentages belonging to physicians and others. Most of the doctors had attended an average of 2 webinars per week. The tests of model effects showed a significant negative correlation of webinar quality ratings for district hospital healthcare setting of the attendees (of *p* value of 0.013) and for the number of COVID-related webinars attended per week (*p* value of 0.009).

**Conclusions:**

Most respondents had favorable perceptions of webinars happening during the pandemic. However, there is a need for improvisation in the volume of webinars, target-audience-based delivery, and participant interaction to add value to this new dimension of teaching-learning.

## Background

The outbreak of highly contagious COVID-19 caused by SARS-Cov2 has caused massive disruption globally. Not only it has resulted in high morbidity and mortality along with economic disruption but has posed an arduous challenge for medical fraternity to continue health services along with continuing medical education and research purposes. The pandemic has challenged almost every sector of today’s world. Health profession and medical care is possibly one of the worst hit field (Infection prevention and control for the safe management of a dead body in the context of COVID-19: interim guidance 2021). The pandemic not only crippled the smooth propagation of on-going clinical care system but also affected the momentum of medical education.

More than 300 million people worldwide has been affected by pandemic till date and educators are striving to ensure that learning continues, in spite of massive disruption (Sleiwah et al. 2020).

Fast and persistent spread of this virus across the world impelled most medical and research institution to adopt and embrace an alternative mode of practice, teaching, and training (Global research on coronavirus disease (COVID-19) 2021).

Online learning has been the best way to reach large number of health care workers (HCWs) and update knowledge. This virtual mode was required to continue the momentum of learning and limit the spread between health professionals which will ultimately keep medical care and education intact.

Lack of face-to-face educational functions and restrictions on academic gatherings has moved us to adopt e-learning using remote working sites. Many of HCW were struggling to re-adjust with new methods but still prepared for longer period of educational ‘social distancing’ (EDEN webinar series: Education in time of a pandemic #onlinetogether #covid19 – Summary 2021).

The online webinars have been designed for these uncertain times, featuring experts in their fields. Each webinar consists of brief presentation addressing topic, further complimented by question-and-answer sessions, where everybody is encouraged to participate and share their experiences. The webinars on COVID have successfully allowed sharing of unbiased information and experiences in a comfortable, multifaceted interactive learning environment, enabling participants to feel connected (Hoke et al. 2017). As webinars require minimal infrastructure, they are shown to be cost effective. With barriers of cost and distance minimized, these have been considered as best tool in widening access to medical education (Power and St-Jacques 2014).

In today’s education system, we have twenty-first century-born people, with unlimited access to information, who use digital technologies on daily basis, and no longer want to passively listen and memorize information served. They expect to leave education system with knowledge and skills that may need to be employable in labor market, to have the capability to be lifelong learners (Developing 21st Century Skills Through Teaching Online: Opportunities and Challenges 2021). Faced with an overnight change in how information about COVID has to be delivered, everybody has to adapt to a new situation, learning new technologies, time management, communication, and elaboration. The challenges in medical education posed by the pandemic have resulted in the increased popularity of alternative teaching-learning methods such as online classes and webinars (Ahmed et al. 2020; Nahai and Kenkel 2020). A number of national and international webinars have been conducted online to overcome the challenges of current pandemic crisis, but were these webinars really worth helpful? Since pandemic has started, everybody is keen to deliver webinars on COVID but how much really are they helping in our educational improvement or management skills has not been evaluated so far. There was need to assess the utility of these webinars in pandemic to HCW so as to actively update the ongoing methods. Hence, this study aimed to evaluate the utility and usefulness of COVID-19-related webinars among HCWs by conducting a questionnaire-based survey. This is the first survey, in our knowledge, to try and evaluate the utility of webinars occurring during the COVID-19 pandemic.

## Methods

A questionnaire-based observational study was conducted after obtaining clearance from the institution’s research review board and ethics committee (NO./INT/IEC/2020/SPL-1402). The study involved the circulation of an online survey, addressing efficacy and usefulness of webinars held during the COVID-19 pandemic. The study population included doctors in age group of 25–75 years who are actively involved in the management of COVID-19 and wish to participate in survey.

A questionnaire-based survey was developed in the English language using Google forms after a brief introduction and informed consent. The survey collected the participant’s personal details and responses to a mandatory questionnaire. The questionnaire was designed to assess information regarding COVID webinars, engagement, and usefulness of webinars. The questionnaire was validated by a local committee of experts in the field. The response to the questions was measured using 4- or 5-point Likert scale.

Voluntary response sampling, a type of non-probability sampling, was used in this study. The link to the survey was circulated through social media (WhatsApp, email). The survey was open for a 4-week period with extension of one week. A reminder to take the survey was sent at the end of 1 week. Personal contact through social media message was also made to improve the response rate. The response link allowed completing the survey only once using a particular email address.

### Statistical analysis

Questionnaire data from 270 respondents was populated from Google sheets into SPSS for analysis. Normality of data was analysed using Shapiro-Wilk test and Kolmogorov-Smirnov test. The descriptive characteristics of the respondents have been presented as median (IQR) for numerical non-normally distributed data, and categorical data have been presented as number and percentage. Each of the six questions, rated on a 5-point Likert scale, was tailored to give us an idea as to whether the webinar style of information dissemination was being found as useful by the end users. The overall usefulness rating was derived from averaging these scores and generalized ordinal logistic regression was conducted with this parameter as the dependent variable. Factors included in the model were gender, professional background, and healthcare setting of the attendees while the covariates included age in years, number of COVID-related webinars attended per week and years of experience in the field. Professional background was categorised as Anaesthesiologists, Intensivist, Physician, and Others. Healthcare setting was categorised as district hospitals and tertiary care centre. Data was analysed using IBM SPSS Statistics for Windows, Version 25.0 (IBM Corp., Armonk, NY., USA, 2017) with RStudio 1.4.1103, for data visualization where needed.

## Results

**Fig. 1:**
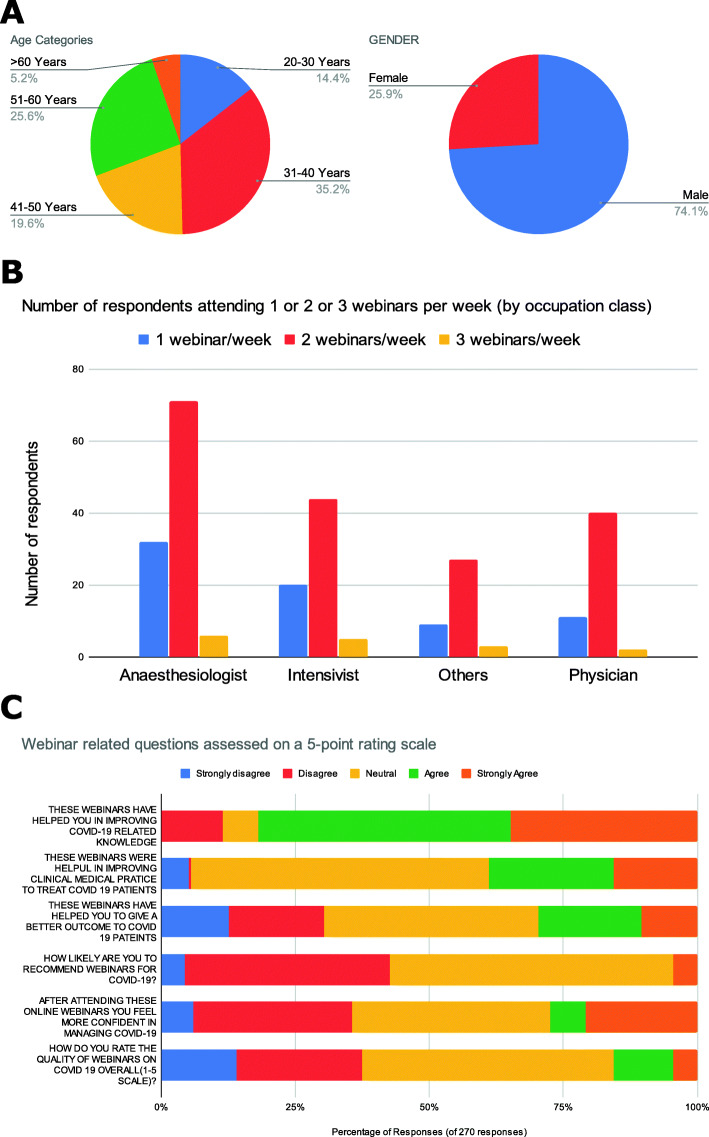
**A** Age and gender distribution in pie charts. **B** Cluster bar chart showing number of attendees in webinars across professional categories split into those attending 1, 2, and 3 webinars per week. **C** Stacked bar chart showing proportion of the different responses to the different questions in our survey

**Fig. 2:**
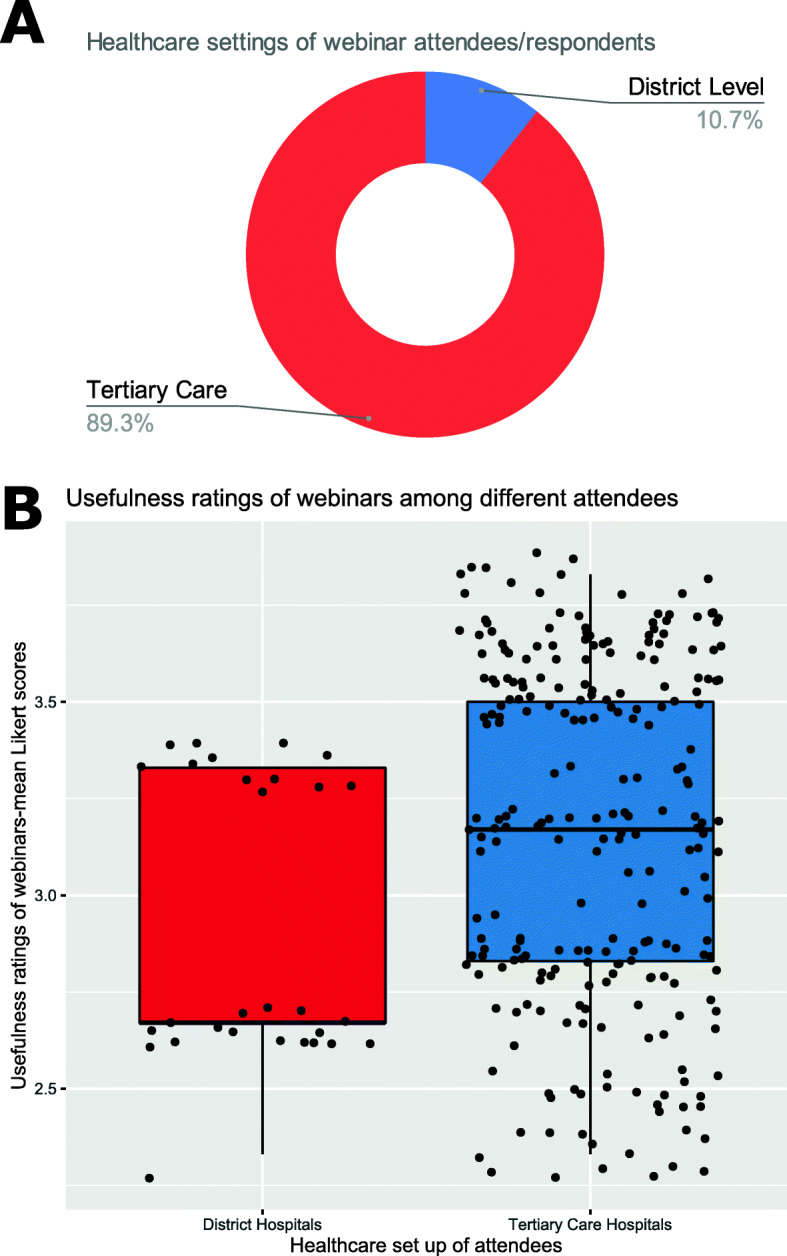
**A** Donut chart showing proportion of respondents from different health care settings. **B** Boxplot with jitter showing distribution of overall usefulness ratings for webinars across the respondents from different health care settings

The response rate was 54% as 270 out of 500 participants responded to the questionnaire. Demographics have been presented in Table 1 and the infographic in Fig. 1 details pie charts showing proportion of respondents with respect to age and gender. Majority were in the 31–40 age group (35.2%) and were males (74.1%). In the same figure a clustered bar chart attempts to display the number of respondents from different professional categories divided into series based on number of webinars attended each week (range of 1–3 webinars per week). It is clear that a major segment of them is from Anaesthesiology and Intensive care and most had attended 2 webinars per week. The lower third of the infographic displays a horizontal stacked bar chart showing the answers to the different questions on a 5-point Likert scale ranging from strongly disagree to strongly agree. A generalized ordinal logistic regression model on inclusion of the factors and covariates showed a significant improvement over the null with a *p* value of 0.011 on the omnibus test. The tests of model effects showed significant *p* values for healthcare setting of the attendees (*p* value of 0.019) and for number of COVID-related webinars attended per week (*p* value of 0.009). Those from district hospital settings (10.7% of total respondents) tended to give a lower usefulness rating for the webinars (Fig. 2) evidenced by their correlation coefficient of − 0.843 (95% CI of − 1.509 to – 0.177) and the greater the number of webinars a person attended in a week the worse they rated the usefulness (correlation coefficient of − 0.538 (95% CI of − 0.945 to − 0.132). The rest of the covariates and factors showed no significant correlation with the overall usefulness rating of the webinars. The lower rating amongst the district hospital respondents could be attributed to the webinars not being tailored to the resources and knowledge base available to them and the poorer rating with increasing number of webinars may be due to repetition of the same information across webinars in the same week. Recording the duration of these webinars might have been a more interesting variable which might help us to tailor future webinars, making them more focused and potentially encourage more information retention. Unfortunately, our survey did not manage to record this variable.

**Table 1:** Characteristics of participants

Parameter	Median IQR or *N* (%)	
Age	42 (32–52)	
Years of experience in field	12 (5–21)	
Number of webinars attended per week	2 (1–2)	
**Age categories**		
> 60 years	14	5.2%
20–30 ears	39	14.4%
31–40 years	95	35.2%
41-50 years	53	19.6%
51–60 years	69	25.6%
**Gender**		
Female	70	25.9%
Male	200	74.1%
**Professional background**		
Anesthesiologist	109	40.4%
Intensivist	69	25.6%
Others	39	14.4%
Physician	53	19.6%
**Health care setting of respondent**		
District level	29	10.7%
Tertiary care	241	89.3%

## Discussion

Regarding the usefulness of webinars, most of the respondents agreed that the webinars had improved their theoretical knowledge regarding COVID-19, helping them to improve their clinical practice. There was a consensus among most respondents that webinars were essential in clinical practice and that they would recommend webinars as a learning modality. This response is consistent with resident’s perception in other fields of medicine that online education should be maintained after the COVID-19 crisis (Kumar et al. 2020; Figueroa et al. 2020). The volume of webinars attended inversely correlated with the perceived quality and usefulness of webinars as a greater number of respondents who had attended ≤ 1 webinars per week responded favorably than those who had participated in > 2 webinars. This dissatisfaction may probably be reflective of the poor quality of webinars or because the same information was being repeated in multiple webinars.

Despite the overall good perception regarding webinars, certain demerits of the webinars have been observed by the attendees. The increased volume and repetition in webinars probably increased boredom. This probably explains why although most felt the webinars were useful many strongly disagreed on recommending the same webinars to others. As compared to one-way passive learning from lectures, webinars can promote interactive learning for the audience. In other studies, the presence of few barriers to e-learning such as poor motivation, concern about the validity of online training, time constraints due to duty hours, and poor technical skills or organizational factors such as the poor design of the webinar and lack of interaction with speakers/trainers tend to hamper the attendees’ acceptance of online webinars as a substantial medium (Assareh and Hosseini 2011; O’Doherty et al. 2018; Rajab et al. 2020). Identification of these barriers was out of the scope of this survey, and further studies may evaluate the same. Apart from these, other possible demerits of webinars that were not assessed by this study include inadequate access to e-learning platform and technical problems related to web connectivity.

The survey indirectly highlights the need to improve the e-learning experience of attendees from webinars. First, the organizations conducting the webinars should coordinate to plan and execute the webinars to avoid repetition of topics and overlap in the timing of multiple events. Second, the target population for the webinar and the webinar’s objectives need to be mentioned in the promotions and invitation links. Perhaps, this will reduce the confusion about which webinars to attend to a certain extent. Third, webinars need to be more interactive.(Carvalho-Silva et al. 2018)^.^ Adding questions and answer sessions to the webinar, dispersed strategically throughout the webinar, rather than only at the end, is one sure way to increase the audience-presenter interaction . A live conversation or chat with the presenters may further improve the experience for the attendees. Multiple other social media platforms such as Twitter, Facebook etc., may be used to extend the interaction beyond the webinars, and the key highlights of the webinar may be posted. A poll at the end to rate the webinar and to review the knowledge gained by attendees, their interests, suggestions, and challenges faced may help organizers in improving webinars in the future.

The study has limitations inherent to most surveys like coverage bias, sampling bias, non-response bias, short duration of the survey, reliability of the questionnaire, and recall bias of the respondents. The reasons for the relatively low response rate in this study may include overlap in the participants in different social media groups, lack of interest to participate in the survey, lack of incentive, and short duration of survey. By taking care of these constraints, the response rate may be improved. Being a cross-sectional survey, the data were collected at a single point in time. However, the attendee’s perceptions regarding webinars may change over time, especially with the transition through the lockdown to the post-lockdown phase.

## Conclusions

We received a positive attitude towards webinars as a method of learning during the COVID-19 pandemic. However, the survey results indirectly highlight the need for improvisation in the current pattern of webinars. The key aspects that may need to be addressed include target-audience-based delivery, uniqueness of the content, and better presenter-attendee interaction. These changes may add value to this newly added dimension of learning, which had been less explored in the pre-COVID era.

## Data Availability

Available on demand.

## Abbreviation

HCW: Health care workers
